# Sensitivity of Buff-Tailed Bumblebee (*Bombus terrestris* L.) to Insecticides with Different Mode of Action

**DOI:** 10.3390/insects13020184

**Published:** 2022-02-10

**Authors:** Guillermo Cabezas, Gema P. Farinós

**Affiliations:** Laboratory of Applied Entomology for Human and Plant Health, Centro de Investigaciones Biológicas Margarita Salas, Ramiro de Maeztu 9, 28040 Madrid, Spain; gcabezastorrero@gmail.com

**Keywords:** pollinators, neonicotinoids, imidacloprid, thiacloprid, pyrethroids, deltamethrin, esfenvalerate, sulfoxaflor, *Bacillus thuringiensis*, Cry1Ab, acute toxicity, bioassays, commercial hives

## Abstract

**Simple Summary:**

Several neonicotinoid insecticides that were once widely used for pest control are currently banned for outdoor use in the European Union (EU) because they pose a risk to bees. This restriction meant that farmers had to look for alternatives for pest management and use known insecticides or new substances with supposedly more bee-friendly characteristics. We evaluated the toxicity of six insecticides on buff-tailed bumblebee workers (*Bombus terrestris)*: two banned neonicotinoids (imidacloprid, thiacloprid), two pyrethroids (deltamethrin, esfenvalerate), one sulfoximine (sulfoxaflor) and a microbial insecticide based on *Bacillus thuringiensis* toxins, which are present in genetically modified (Bt) maize. The results obtained show that certain insecticides in use have higher acute toxicity to *B. terrestris* than some of the banned neonicotinoids.

**Abstract:**

Systemic insecticides are recognized as one of the drivers of the worldwide bee decline as they are exposed to them through multiple pathways. Specifically, neonicotinoids, some of which are banned for outdoor use in the European Union (EU), have been pointed out as a major cause of bee collapse. Thus, farmers have had to look for alternatives for pest control and use known insecticides or new substances reportedly less harmful to bees. We evaluated the oral acute toxicity of six insecticides (three of them systemic: imidacloprid, thiacloprid and sulfoxaflor) with four different modes of action on buff-tailed bumblebee workers (*Bombus terrestris*): two banned neonicotinoids (imidacloprid, thiacloprid), two pyrethroids (deltamethrin, esfenvalerate), one sulfoximine (sulfoxaflor) and a microbial insecticide based on *Bacillus thuringiensis* toxins, present in genetically modified (Bt) maize. The microbial insecticide only caused mortality to bumblebee workers at extremely high concentrations, so it is expected that Bt maize does not pose a risk to them. The toxicity of the other five insecticides on bumblebees was, from highest to lowest: imidacloprid, sulfoxaflor, deltamethrin, esfenvalerate and thiacloprid. This outcome suggests that certain insecticides in use are more toxic to *B. terrestris* than some banned neonicotinoids. Further chronic toxicity studies, under realistic conditions, are necessary for a proper risk assessment.

## 1. Introduction

The genus *Bombus* Latreille, with a wide world distribution that includes Europe, Asia, North America, parts of South America and North Africa [1], is highly efficient for pollinating not only a wide variety of wild plants but also crops [2,3]. Within this genus, the *Bombus terrestris* L./*Bombus lucorum* L. complex is the first in the ranking list of the most important crop pollinators in Europe, with a mean contributed value of USD 425 per hectare [4]. The different subspecies of *B. terrestris* are originally distributed throughout Eurasia and North Africa [5], and their characteristics as pollinators, together with the relative ease of rearing them in captivity, have led to their domestication for use in the pollination of horticultural, fruit and seed crops, with particular importance in tomato and berry greenhouses [2,3]. 

Since the mid-1980s, a severe decline in managed honey bee populations has been observed in Europe and the US [6,7,8,9]. Furthermore, where evidence is available, such as the UK, Netherlands, Belgium or the US, it has been demonstrated that the abundance and diversity of non-*Apis* bees, including bumblebees, were also being affected [6,10,11,12,13,14]. Although there is no single cause responsible for this decline, agricultural intensification has been cited as one of the most important threats to pollinators [15,16]. Pesticides (and particularly insecticides) have been the subject of debate ever since the collapse in bee populations was observed [17], although the neonicotinoids are the ones that have mainly been in the focus for years. Since their launch in the 1990s, neonicotinoids have become one of the most widespread classes of insecticides globally, to such an extent that in 2014 they accounted for 25% of the insecticide market [18]. This success has been mainly due to their effectiveness in controlling a number of important pests and their versatility in application (soil and seed treatments and foliar spray) [19]. One of their most valuable characteristics is their systemic nature, which allows them to reach all plant tissues and organs. However, it is precisely their systemicity, together with their toxicity on invertebrates, persistence and environmental impact, that makes them potentially harmful to a wide range of non-target organisms [20]. 

Various laboratory and semi-field trials have reported that dietary neonicotinoids produce harmful sublethal effects on honey bees and non-*Apis* bees [21,22,23,24]. These negative effects have also been reported in different studies conducted under field conditions, in which bumblebees and solitary bees were exposed to several neonicotinoids by different routes, leading to important sublethal effects, such as reduced solitary bee nesting and reproductive success and bumblebee colony growth and reproduction [25,26,27]. Nowadays, systemic insecticides are recognized as one of the drivers of worldwide honey and wild bee declines as they are exposed to them when they collect crop pollen and nectar [28,29,30]. Despite the large number of studies conducted to assess the effects of neonicotinoids on bees, numerous gaps have been identified in the methodology used and the subjects studied in relation to this issue, and further research using species other than the honey bee *Apis mellifera* L. has been recommended to improve laboratory, semi-field and field tests [31,32,33]. 

In 2013, in the light of scientific and technical evidences, the European Commission considered that the active substances clothianidin, thiamethoxam and imidacloprid posed a high risk to bees, so restrictions on their use were imposed in the European Union (EU), mainly affecting maize, sunflower and oilseed rape [34]. Due to extensive evidence from scientific research on the detrimental effects of neonicotinoids on bees, in May 2018 the Commission definitively banned the outdoor use of these three neonicotinoids based on risk assessment and scientific data [35,36,37]). This ban meant that farmers had to look for alternatives for pest management. The first option was to replace them with other neonicotinoids with a similar mode of action, such as acetamiprid or thiacloprid, the latter mainly applied to seed in the case of maize and sunflower, or by foliar spray in sunflower and rapeseed [38]. However, an application to renew the approval of thiacloprid was rejected by the European Commission in January 2020. Another option was to replace the banned neonicotinoids with other broad-spectrum and widely used chemical insecticides, mainly pyrethroids [38,39]. A third avenue for the replacement of neonicotinoids was to explore promising new active substances that are supposedly less aggressive towards non-target pests. One example is the active substance sulfoxaflor, belonging to the sulfoximine family, which was authorized in 2015 in the EU. It is a systemic insecticide with translaminar movement and acts mainly by ingestion and contact. It has been proposed as an alternative to neonicotinoids given its lower toxicity to bees and better ecological and human health profile, among other reasons [40,41]. 

In addition to insecticides sprayed on crops, bumblebees may also be exposed to other insecticidal substances, such as the toxin Cry1Ab from *Bacillus thuringiensis*, expressed in genetically modified maize varieties derived from the MON810 event (Bt maize). It has been found that maize pollen can be attractive for honey bees during dry growing seasons or periods when more favorable protein sources may not be available [42,43]. Furthermore, in agricultural landscapes with large-scale monocultures, pollen foragers might be forced to almost exclusively collect pollen from a single source, even from wind pollinated crops such as maize [44]. Thus, in areas where its level of adoption is high, exposure to Cry1Ab protein could represent a risk to bees. It has been demonstrated that the pollen from Bt crops can contain significant amounts of Cry toxin, although the toxin levels found differed depending on the study [45,46,47]. Information on the toxic effects of Cry1Ab toxin on bees is scarce and sometimes contradictory. Some studies indicate that it does not cause acute or chronic toxic effects on honey bees (*A. mellifera*) or bumblebees [46,48]. However, it has been shown that it can cause sublethal effects on honey bees by affecting their foraging behavior or feeding [49,50]. 

The aim of this study is to evaluate the oral acute toxicity of six insecticides with four different modes of action on the bumblebee *Bombus terrestris*: two neonicotinoids (imidacloprid and thiacloprid), two pyrethroids (deltamethrin and esfenvalerate), one sulfoximine (sulfoxaflor) and a microbial insecticide based on Cry toxins from *Bacillus thuringiensis*. The possible implications of the results obtained, following the restriction of the use of neonicotinoids in the EU, are discussed.

## 2. Materials and Methods

### 2.1. Maintenance of the Hives

Workers of the buff-tailed bumblebee *B. terrestris* were used to carry out the bioassays with insecticides. The workers came from hives supplied by the company Agrobío S.L (Almería, Spain), consisting of a fertilized queen, 20–40 workers and a nest with larvae and eggs. Twenty-one commercial hives were used to perform the bioassays (Table S1). Once in the laboratory, they were kept in a chamber at a constant temperature of 25 ± 1 °C, relative humidity of 60 ± 5% and in conditions of complete darkness, illuminating the chamber with red light only when it was necessary for handling or for picking up individuals. The bees were provided ad libitum with syrup (Api 65^®^, Agrobío S.L., Almería, Spain) and food balls, prepared with dry pollen, artificial syrup and distilled water. Workers for the bioassays were extracted at the moment of exponential growth of the hive.

### 2.2. Insecticides

We selected six different active ingredients for bioassays: (i) two neonicotinoids, imidacloprid (Confidor^®^ SL 20% *W/V*, Bayer S.A.S., Lyon, France) and thiacloprid (Calypso^®^ SC, 48% *W/V*, Bayer CropScience, S.L., Valencia, Spain), both currently restricted in the EU; (ii) two pyrethroids, deltamethrin (Decis Protech^®^ EW, 1.5% *W/V*, Bayer CropScience S.L., Valencia, Spain) and esfenvalerate (Sumifive^®^ Plus EW, 5% *W/V*, Kenogard, S.A., Madrid, Spain), belonging to a new generation of pyrethroids, which show a higher effectiveness than the previous ones and widely used on a large number of crops for the control of different pests; (iii) the sulfoximine-based pesticide sulfoxaflor (Closer^®^ SC EW 11.43% *W/W*, Dow Agrosciences Iberica S.A., Seville, Spain) for being a relatively new systemic insecticide, proposed as a candidate for replacement of the neonicotinoids [51]; (iv) the selective biological insecticide *Bacillus thuringiensis* ssp. *kurstaki* (Btk) (DiPel^®^ DF WG, 54% *W/W*, strain ABTS-351 (32 mill. CLU/g), Kenogard, S.A., Valent BioScience LLC, Libertyville, IL, USA), containing four insecticide Cry toxins, one of which (Cry1Ab) is expressed in the genetically modified maize cultivated in the European Union. All insecticide concentrations were prepared in Api 65^®^ artificial syrup with the exception of the insecticide DiPel^®^ DF, which was previously diluted in 0.1% Triton^®^ X-100 (Sigma-Aldrich, St. Louis, MO, USA).

### 2.3. Bioassays 

The methodology used to carry out the bioassays was almost entirely in line with that recommended for studying the acute oral toxicity of insecticides in bumblebees, with some modifications [52]. Concentration–response bioassays were conducted on individualized *B. terrestris* workers. Each bioassay consisted of treating individual workers with 5–7 increasing nominal test concentrations of insecticide diluted in 2 mL of artificial nectar. The range of nominal test concentrations of each of the insecticides used in the bioassays was decided after preliminary trials in order to obtain a response (mortality) between 10% and 90%. Once the extreme concentrations were defined, intermediate concentrations were determined on a logarithmic scale. A negative control was added consisting of 2 mL of artificial nectar without insecticide treatment and a positive control consisting of 2 mL of artificial nectar treated with 80 ppm (*W/V*) of the pyrethroid lambda-cyhalothrin (Karate Zeon^®^ 10% *W/V* CS Syngenta S.A., Madrid, Spain), previously known to be toxic to bumblebees [53]. 

Bees between 1 and 4 days old were selected for the bioassays. Newly emerged workers, distinguishable by the absence of pigmentation and the shape of the wings yet to unfold, were removed from the hives and separated into groups of five bees of similar size and same hive of origin. They were placed in round transparent plastic boxes (11.5 cm diameter, 5 cm high and 450 mL capacity) with a lid fitted with a ventilation grid and the base covered with filter paper. Each box contained a jar (2.5 cm in diameter, 1.3 cm high and 3 mL capacity) with pollen similar to that used to feed the hives, and another jar with absorbent cotton impregnated with Apis 65^®^ artificial nectar (Agrobío S.L., Almería, Spain). The bees were kept in these conditions for 24 h. After this time, their condition was evaluated to assess their inclusion in the bioassay, using only those that were in optimal conditions. We evaluated whether there was a relationship between the weight of the bees and the volume of artificial nectar consumed, which could affect the outcome of the bioassays. For this purpose, 40 bees from 10 different hives were selected and fed for 48 h with untreated nectar. After this time, the bees were weighed and the weight of food consumed per bee was calculated. Once the weights of the bees were known, the weight of the workers used in the bioassays was standardized for each of the hives. Thus, those bees whose weight was not within 2 standard deviations of the mean weight were discarded.

The bioassay was performed by placing individual bees, previously anaesthetized with CO_2_ for 10 s, in queen cages to which an empty 10 mL plastic syringe was attached. The bees were starved for 3 h. After this period they were weighed with a balance (LPW-523i, VWR, Milan, Italy) to determine their initial weight and the empty syringes were replaced with pre-weighed syringes containing the different treatments. To enable the treatment to be taken, the tip of the syringes was cut off, and to ensure that the liquid was accessible throughout the bioassay, they were placed with a slight inclination. Once the treatment syringe was attached, each bee was checked for feeding. In each bioassay, between 2 and 6 replicates were carried out, where a replicate is defined as the set of treated bees from each commercial hive. At least 4 bees from the same source hive were assigned to each of the concentrations and controls.

The bioassays lasted for 48 h or until the bees died. The condition of each worker bee at 2, 4, 24 and 48 h after the start of the bioassay was assessed according to the responses to mechanical stimuli (gentle blows on the cage containing the bee) and light stimuli (illuminating each bee with white light for a few seconds). The bees were given a numerical value according to the following scale: 0 (dead bee that does not react to any stimulus), 1 (bee in ataxia state that does not move, but abdominal or limb movements are observed), 2 (bee that moves awkwardly but can access the food source) and 3 (bee that is in an optimal state and is able to move without difficulty through the cylinder and reach the food source). After 48 h, or when the bees died, they were reweighed. For mortality purposes, bees whose status was 0 and 1 at the end of the bioassay were taken into account. 

To determine how much the workers had consumed in each of the treatments, the syringes with the unconsumed treatment inside were reweighed when the bees died or at the end of the bioassay. In this way, the weight of the consumed treatment was calculated by subtracting the final weight of the syringes from the initial weight. The value obtained was corrected for the weight loss of the treatments caused by evaporation. To calculate this weight loss, 3 to 10 syringes containing 2 mL of each insecticide concentration, as well as both controls in each bioassay, were attached to empty queen cages and kept in the same conditions and for the same period of time as the bioassay syringes, and the mean value of weight lost due to evaporation was subtracted from the consumption value. To obtain the daily consumption/bee, the corrected weight of treated nectar consumed was divided by the number of days the bee was able to access the food source (stages 2 and 3 of the numerical scale).

### 2.4. Maximum Exposure of Bumblebee Workers in the Field

Numerous studies carried out with different methodologies have identified a large number of pesticide residues in pollen, nectar and honey samples. Taking into account the available information, we estimated the maximum dose to which bumblebee workers could be exposed in the field in a worst case scenario. For this purpose, we used values of maximum daily nectar consumption of bumblebee workers in our bioassays (554 mg/bee/day) and values of the maximum concentration detected in nectar or honey of each of the active substances evaluated in the bioassays [54,55]. In the case of esfenvalerate, as no published data on residues in nectar or honey were found, the value of fenvalerate residue in honey was taken, as esfenvalerate is an isomer (2S, αS) of fenvalerate.

### 2.5. Statistical Analyses

To test whether there was a relationship between the weight of the workers and the amount of food ingested in *B. terrestris*, a Pearson correlation was performed with the values of the weight of the workers after feeding on nectar for 48 h and the amount of nectar consumed by each one of them. SPSS (IBM© SPSS© Statistics, Version 25.0, 2017) was used to perform this analysis, and a significance level of α = 0.05 was set.

Mortality data from the concentration–response bioassays for each of the insecticides were analyzed by probit analysis. The PoloPlus program (LeOra Software, 2002–2019) was used, which calculates a concentration–response regression line on a logarithmic scale. To determine the susceptibility of *B. terrestris* workers to the different insecticides, the LC_50_ and LC_90_ values (concentration that causes mortality at 50% and 90% of the population, respectively), as well as their 95% confidence intervals, were calculated for each of the insecticides. The dose (µg active ingredient/bee) was calculated taking into account the daily amount of insecticide-treated nectar consumed by each bee. Once the insecticide doses were obtained, the LD_50_ and LD_90_ values were calculated (dose that causes 50% and 90% mortality in the population, respectively) together with their 95% confidence intervals. Significant differences in the susceptibility of workers to the different insecticides were tested using the 95% confidence intervals of the lethal concentration ratio (LCR) and lethal dose ratio (LDR) at the LC_50_ and LD_50_ point, respectively. Confidence intervals including value 1 indicate that there are no significant differences between the values compared [56]. 

The survival rate of the workers at the different applied concentrations of each insecticide was calculated using the Kaplan–Meier method, and the differences between the survival probabilities at each concentration were analyzed using the Mantel–Cox log-rank test, setting a significance level of α = 0.05. The probability of survival (%) was defined as the probability that an individual survives longer than time “t”. These analyses were carried out with GraphPad Prism 5.01 software.

## 3. Results

### 3.1. Relationship between Worker Weight and Nectar Consumed

**Figure 1:**
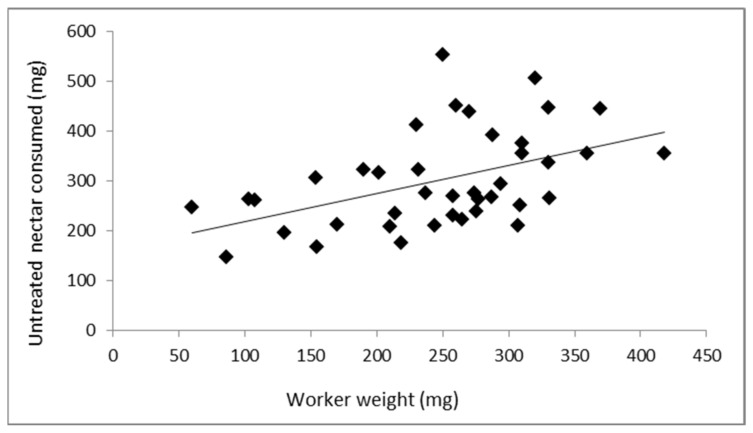
Correlation between the weight of *B. terrestris* workers fed with untreated artificial nectar and the amount of food consumed.

Forty workers with a mean (±SE) weight of 247.4 ± 12.8 mg (range: 60–418 mg) were used to evaluate the relationship between the bee weight and the amount of food consumed. They consumed an average of 302.6 ± 15.2 mg of artificial nectar in 48 h. Pearson’s correlation showed a moderate positive relationship between both variables (r = 0.474; *p* = 0.002), indicating that nectar consumption was higher with increasing bee size (Figure 1). Therefore, the acute toxicity of the insecticides was evaluated by selecting workers of similar size within each hive.

### 3.2. Intake of Treated Artificial Nectar 

**Figure 2:**
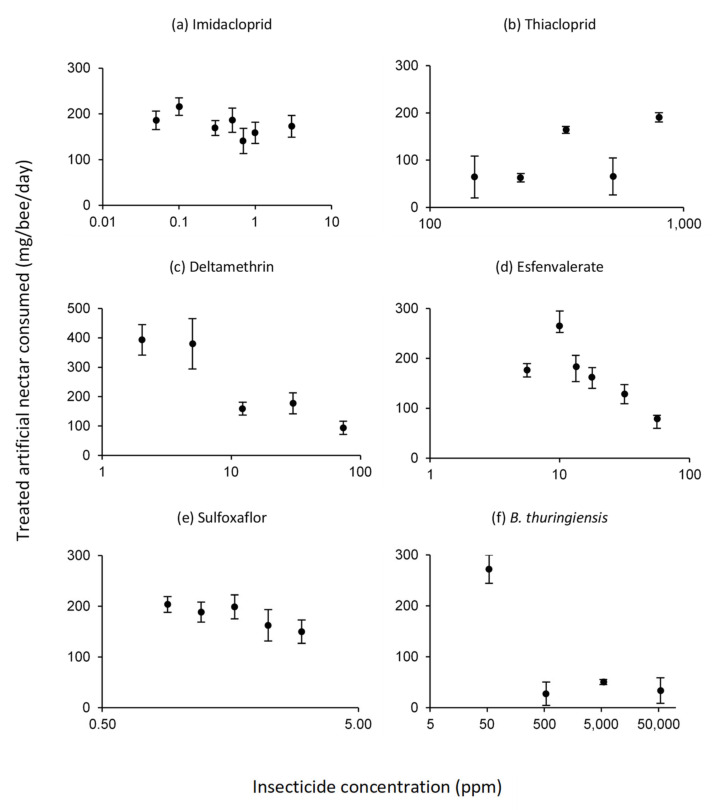
Average daily consumption per bee of artificial nectar treated with different concentrations of the insecticides imidacloprid (**a**), thiacloprid (**b**), deltamethrin (**c**), esfenvalerate (**d**), sulfoxaflor (**e**) and *B. thuringiensis* (**f**). Values expressed in mg of nectar consumed/bee/day. Average values of untreated nectar consumed daily by control bees in each bioassay are: 153.1 ± 20.3 (imidacloprid), 234.7 ± 44.3 (thiacloprid), 466.8 ± 31.5 (deltamethrin), 204.9 ± 13.9 (esfenvalerate), 177.6 ± 15.5 (sulfoxaflor) and 225.1 ± 17.3 (*B. thuringiensis*).

We observed a hive effect in the amount of nectar consumed by worker bumblebees. The values of untreated nectar consumption of the controls ranged from 150 to 250 mg, except for the workers used in the deltamethrin bioassays, whose consumption was almost double (467 ± 31) (Figure 2). In general, the intake of insecticide-treated nectar was irregular, although with the pyrethroids and sulfoxaflor a trend of decreasing consumption was observed as the concentration of insecticide in the nectar increased (Figure 2). Workers exposed to nectar containing thiacloprid showed a drastic reduction in nectar ingestion compared to controls. Likewise, a very sharp decrease in insecticide consumption of artificial nectar treated with *B. thuringiensis* was observed at the three highest concentrations of the four tested (Figure 2), which in this case corresponded to those used in the preliminary trial.

### 3.3. Toxicity of Insecticides on Bumblebee Workers

In all bioassays of the six insecticides tested, the mortality of the positive controls was 100%, while no bees died in the negative controls, except in the case of sulfoxaflor where there were two dead bees (9.1% mortality). A total of 149 bees weighing 240.5 ± 8.9 mg from five commercial hives were fed for 48 h with concentrations of the neonicotinoid imidacloprid ranging between 0.05 and 3 ppm. Fitting the mortality data to the probit model resulted in an LC_50_ of 0.38 (0.22–0.76) ppm and LD_50_ of 0.13 (0.08–0.24) µg a.i./bee (Table 1). For thiacloprid, 95 workers weighing 225.2 ± 7.9 mg extracted from two hives were treated with increasing concentrations of thiacloprid, from 150 to 800 ppm. The toxicity of this insecticide to *B. terrestris* workers gave values of LC_50_ of 424 (296–815) ppm and LD_50_ of 90.5 (58.8–172.7) µg a.i./bee (Table 1). The deltamethrin bioassay was conducted with 99 workers with an average weight of 251.8 ± 8.1 mg from two commercial hives. The concentrations used ranged between 2.04 and 73.53 ppm, and mortality resulted in LC_50_ of 7.1 (3.3–11.9) ppm and LD_50_ of 3.65 (2.19–5.11) µg a.i./bee (Table 1). Seven commercial hives were used to carry out the bioassay with the insecticide esfenvalerate, with a total of 191 bees weighing on average 205.4 ± 5.6 mg. The concentrations used for this insecticide were between 5.6 and 56.2 ppm, and its toxicity gave values of LC_50_ of 17.8 (14.4–22.4) ppm and LD_50_ of 5.52 (4.55–6.70) µg a.i./bee (Table 1). A total of 126 workers with a mean weight of 245.2 ± 5.3 mg from five hives were treated with increasing concentrations of sulfoxaflor ranging between 0.9 and 3 ppm. Fitting the mortality data to the probit model resulted in an LC_50_ of 2.22 (1.76–3.85) ppm and LD_50_ of 0.71 (0.56–1.01) µg a.i./bee (Table 1). In all cases with these five insecticides, the χ^2^ values of the probit analyses performed for the calculation of the LC and LD values did not exceed the tabular values corresponding to the degrees of freedom in each case, indicating a good fit of the models. 

Four commercial hives were used to carry out the bioassays with the microbial insecticide *B. thuringiensis*, with a total of 94 bumblebee workers weighing 267.9 ± 5.3 mg. The range of concentrations used in the preliminary bioassay was between 54 and 54,000 ppm, the latter being the maximum concentration that could be prepared with the commercial insecticide DiPel^®^ DF. It was not possible to fit the mortality data to the probit model because responses between 10% and 90% were not achieved. Mortality was only recorded in the group of workers treated with the 54,000 ppm concentration, which caused 73.7% mortality.

### 3.4. Survival Probability after Insecticide Treatment

**Figure 3:**
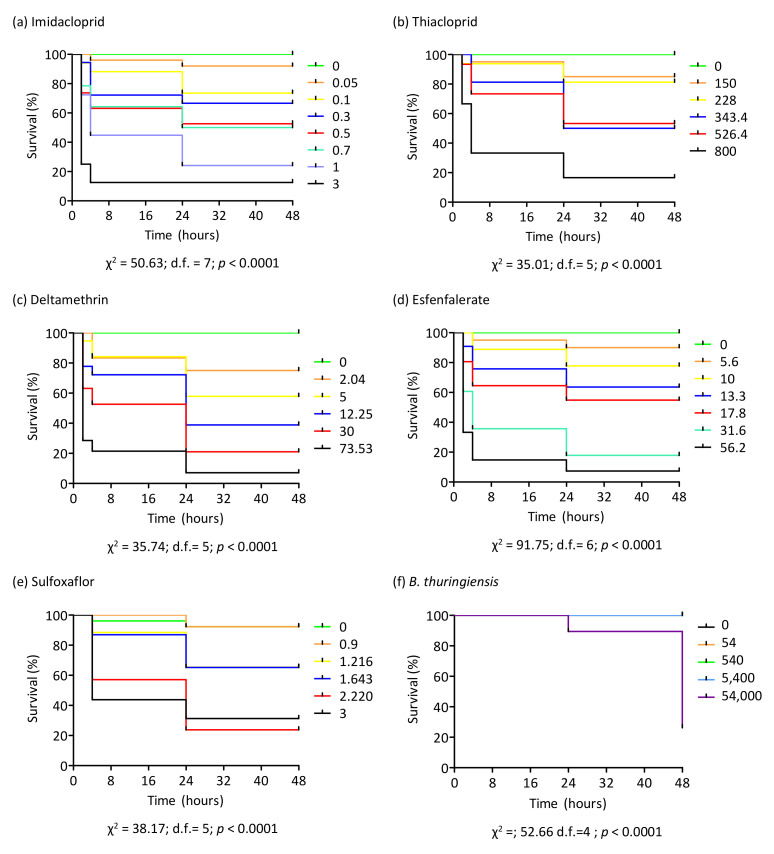
Kaplan–Meier survival analyses for *B. terrestris* workers when they were exposed for 48 h to different concentrations of the insecticides imidacloprid, thiacloprid, deltamethrin, esfenvalerate, sulfoxaflor and *B. thuringiensis*. Concentrations are represented by different colors and are expressed in ppm (*W/V*). The probability of survival (%) is defined as the probability that an individual survives longer than time “t”.

The Kaplan–Meier survival test was performed for the six insecticides tested. The probability of survival was below 20% by 24 h after starting treatment with the highest concentrations tested, except for sulfoxaflor and *B. thuringiensis*. In these two cases, the survival probability with all concentrations was higher than 20% at the end of the treatment (48 h) (Figure 3).

### 3.5. Maximum Exposure of Bumblebee Workers in the Field

Of the five insecticides studied, neonicotinoids were found to be those to which *B. terrestris* workers were most exposed (first thiacloprid and secondly imidacloprid, with 115.7 and 40.3 ng a.i./bee/day, respectively), taking into account the residues found in nectar and the amount of nectar they can ingest (Table 2), followed by sulfoxaflor and deltamethrin. Taking into account the LD values obtained in our bioassays, with imidacloprid a bumblebee worker would need only 3.2 days of maximum ingestion of the highest concentration of insecticide found in the residue (worst case scenario) to reach the LD_50_ value (0.13 µg a.i./bee). This result contrasts with 92.9 days to reach the corresponding LD_50_ value in the case of sulfoxaflor, and almost 800 days for the other neonicotinoid, thiacloprid. Pyrethroids showed the longest time to reach the LD_50_ values in a worst case scenario. (Table 2). 

## 4. Discussion

We have evaluated the oral acute toxicity of six insecticide active substances in the buff-tailed bumblebee *B. terrestris*, one of most economically important pollinator species. Of the insecticides evaluated, both neonicotinoids are currently restricted for use in the EU (imidacloprid since 2018 and thiacloprid since 2021), and the rest (two pyrethroids, sulfoxaflor and *B. thuringiensis*) are used on many crops in the EU for the control of a wide range of pests.

The intake of insecticide-treated nectar did not show a dependence on the concentration used, except in the cases of deltamethrin and sulfoxaflor. With the two pyrethroids evaluated, deltamethrin and esfenvalerate, a trend towards reduced feeding with increasing insecticide concentration was observed. This could be an indication of a repellency effect, such as it was observed with deltamethrin in *B. terrestris* [57], or with alpha-cypermethrin and lambda-cyhalothrin in *A. mellifera* [58]. However, apart from a possible deterrent effect, it could also be an indication that the insecticide in the diet reduces the bees’ ability or need to forage [59], which cannot be ruled out. Some studies have suggested that some neonicotinoids, such as imidacloprid, may have an antifeedant effect on *B. terrestris* [22,60]. On the contrary, two-choice feeding assays showed that *B. terrestris* preferred to eat more of sucrose solutions laced with the neonicotinoids imidacloprid and thiametoxam than sucrose alone [61]. Our results with the insecticides imidacloprid and thiacloprid do not match either of these two cases, as no concentration-dependent reduction or increase in consumption was observed. 

The results obtained with both LC_50_ and LD_50_ reveal that the acute toxicity levels of the different insecticides that caused mortality to bumblebees followed the following sequence: imidacloprid > sulfoxaflor > deltamethrin > esfenvalerate > thiacloprid. Particularly remarkable is the large difference in toxicity found between the two neonicotinoids. The differential toxicity observed in these insecticides within the same class (almost 700 times more toxic imidacloprid than thiacloprid in terms of LD_50_ and 275 times in terms of LC_50_) were likewise noticed for buff-tailed bumblebees in other studies where LD_50_ of imidacloprid resulted in being 518 times lower than that of thiacloprid [62,63]. Similar results had also been obtained previously in *A. mellifera*. In this species, the acute toxicity of imidacloprid expressed in LD_50_ was 884 [64] or 815 [65] times higher than thiacloprid, or six times more toxic considering LC_50_ values [66]. The differences observed in bee toxicity between the two neonicotinoids tested seem to be caused by the different chemical structure of these two compounds, despite having the same mode of action. Thus, the nitro-substituted neonicotinoids (clothianidin, dinotefuran, imidacloprid and its metabolites, thiamethoxam, nitenpyram) are much more toxic to bees than the cyano-substituted neonicotinoids (acetamiprid and thiacloprid), which exhibit a much lower toxicity [67]. In addition, in both honey bees and bumblebees, it has been found that sensitivity to nitro- and cyano-substituted neonicotinoids are associated to differences in their detoxification by P450 enzymes, demonstrating that both pollinators have biochemical defense systems that define their sensitivity to insecticides [62]. The results of our experiment with imidacloprid showed a somewhat lower survival probability than previously observed in *B. terrestris* workers, which was 75% after four days of oral exposure at 0.1 ppm [60]. However, in our study, the probability of survival was about the same only two days after an exposure at that concentration, so a lower survival would be expected if the treatment had been continued for two more days.

Typically, acute toxicity risk is considered for direct oral or contact exposure of bees to insecticides due to spray drift. However, an important part of the toxicity to which bees are subjected in crop fields comes from the insecticide residues to which they are exposed through multiple pathways, not only within the crop but also in the surrounding area [68,69,70]. In general, residues can be found in pollen and nectar after spray treatments [54]. Bees may also be exposed to residues through guttation droplets, as is the case with neonicotinoids in maize crops [71,72], and by dust drift from sowing treated seeds, especially significant in many neonicotinoids [73]. Neonicotinoids, as systemic insecticides, can be present in nectar and pollen, so it is easy for pollinators to be exposed to them [74,75]. Thiacloprid is in residues much more than imidacloprid [54,76]. Our results show that the maximum exposure dose of bumblebee workers to thiacloprid in a worst case scenario is almost three times higher than that of imidacloprid. Despite this, as thiacloprid has a much lower acute oral toxicity, it can be observed that in this scenario, a worker would need about 800 days to reach the LD_50_ value after maximum ingestion of contaminated nectar, compared to 3 days for imidacloprid. 

Despite the differential toxicity shown by imidacloprid and thiacloprid, as well as their different residual presence in pollen and nectar, there were various reasons related to their environmental risk that led to the banning of both in the EU. Following their withdrawal, it is likely that other insecticides, particularly pyrethroids, will take their place in agricultural pest control [38,39]. We have found that two of them, deltamethrin and esfenvalerate, have intermediate toxicity on bumblebee workers, between the two neonicotinoids, in terms of LC_50_ and LD_50_ values, although our deltamethrin LD_50_ result was higher (lower toxicity) than a previous observation with the same product [57]. All pyrethroids have been found to be potentially toxic towards *B. terrestris* [77]. On the other hand, pyrethroids are lipophilic insecticides and very easily degraded in the natural environment. This characteristic means that many of them, such as esfenvalerate, are not normally present in nectar or honey after field treatments at recommended dosages [54,76]. We used deltamethrin and fenvalerate to evaluate the maximum exposure to these insecticides in a worst case scenario, and we found that it was very low for bumblebee workers, indicating that the nectar residue levels of these pyrethroids in nectar would be below the acute toxicity levels for *B. terrestris*. This result coincides with that found in *A. mellifera*, where it was observed that despite the presence of insecticide residues in honey and nectar, these were below the acute oral toxicity for this species [76]. If pyrethroids are finally the insecticides that will mainly replace neonicotinoids, it is important that acute oral toxicity studies on buff-tailed bumblebee are complemented by other studies of contact toxicity, chronic exposure and effects on larvae, as well as possible synergies and/or additive effects with other substances. Chronic treatment with the pyrethroid λ-cyhalothrin resulted in *B. terrestris* workers with a significantly lower body mass, although this did not affect the reproductive output of colonies or survival [78]. However, chronic dietary λ-cyhalothrin exposure caused severe decreases in survival, food consumption and reproduction of the same species [79]. Moreover, chronic exposure of bumblebees to imidacloprid and λ-cyhalothrin at concentrations that could approximate field-level exposure affected foraging behaviour and increased worker mortality, leading to significant reductions in brood development and colony success [53]. Available information indicates much variability in the toxicity of pyrethroids, which depends on the study subject, the active ingredient used and the type of bioassay performed to assess toxicity, so that risk assessment should be analyzed on a case-by-case basis. In addition, from the point of view of environmental and health risks, it should be considered that, sometimes, pyrethroid metabolites or photodegradates are just as or more harmful than parent compounds for non-target organisms, including mammals and the environment [80].

The sulfoximine sulfoxaflor was the second most toxic insecticide to buff-tailed bumblebee workers of the six tested in terms of LC_50_ and LD_50_, behind only imidacloprid. It belongs to the group of competitive modulators of the nicotinic receptor of acetylcholine, and it is a systemic insecticide that acts mainly by ingestion and contact but with lower toxicity to bees, so it was proposed as an alternative to neonicotinoids [40]. The data available so far on its effects on *B. terrestris* seem to indicate that chronic exposure of colonies to sulfoxaflor may have important sublethal effects, such as reduction in egg laying and larval and worker production, leading to fewer reproductive offspring [51,81], which may also be aggravated by different stressors [82]. A recent study found sulfoxaflor to be about ten times more toxic after 48 h treatment than we observed [83]. However, our results are consistent with those of this study, which suggests that sulfoxaflor was less toxic than imidacloprid for *B. terrestris* but more toxic than the recently banned thiacloprid. Sulfoxaflor has also been found to appear residually in nectar or honey after field treatments, although in smaller quantities, in general, than neonicotinoids [54,55], but it is highly soluble, allowing it to reach wild plants near the treated crop, which could pose a risk factor for visiting pollinators [84]. We have found that the maximum exposure dose of sulfoxaflor for bumblebees in a worst case scenario is higher that the two pyrethroids but lower than the neonicotinoids. It could then be considered that it is a relatively safe insecticide for pollinators as far as its presence in nectar residue is concerned, but its sublethal effects after chronic exposures should be carefully considered. Despite this, in the USA, it is used in a large number of crops for the control of different pests, and, recently, the Environmental Protection Agency has announced that it poses a lower risk to non-target wildlife, including pollinators, than other registered alternative products if used according to the label [41].

The microbial insecticide formulated from the Cry insecticidal toxins of *B. thuringiensis* was the only one of the six insecticides tested with which no acute toxicity was observed in buff-tailed bumblebee workers. Nectar consumption was 5–7 times lower in the three highest concentrations tested (540, 5,400 and 54,000 ppm) than in the control, most likely due to the texture resulting from the dilution of the insecticide, which considerably thickened the treated artificial nectar, and this low consumption could have affected the toxicity results. Even so, workers were only affected at the highest concentration. Although the quantity of nectar consumed was small, there was nectar intake in all three concentrations, comparable to the amount of treated nectar consumed at certain concentrations in thiacloprid, deltamethrin or esfenvalerate bioassays, where the probability of the survival of workers was much below 50% after 48 h (Figure 3). Thus, if Bt were toxic, it would be at significantly lower levels than the other insecticides because we did not find differences in the probability of survival with respect to the control. Given the results of nectar intake in this bioassay, the commercial formulation of Bt used is not appropriate, so it would be convenient to use other sources of Cry toxin to make a more accurate assessment of its toxicity. Spain is the European leading country in the cultivation of maize expressing the insecticidal Cry1Ab toxin of *B. thuringiensis* (MON810 Bt maize), with 98,152 ha grown in 2020 (95% of the total in the EU). This area of Bt crop could pose a problem for pollinators visiting it. Even though maize does not require pollinating insects, they may occasionally use this pollen as a resource [42,43,44], thus being exposed to the insecticidal protein. However, it has been determined that pollen is the maize tissue in which the lowest levels of the Cry1Ab insecticidal toxin is found [45,47]. On the basis of the levels of Cry1Ab measured by these authors, it follows that the maximum concentration we have tested is about 10^5^ times more concentrated than the maximum concentration of toxin detected in the pollen of MON810 maize grown in the EU. Thus, Bt maize would not be expected to be a source of toxicity for *B. terrestris*. Toxic effects have also not been found in bumblebee workers when eating Bt maize pollen [50] or following chronic exposure to Cry1Ab toxin [85]. Nevertheless, Bt maize could represent a cumulative/synergistic stressor since there are evidences that Bt toxins can pose sublethal effects to honey bees by affecting their foraging behavior [49], so further studies under realistic Bt maize field conditions are needed to confirm our findings.

## 5. Conclusions

The withdrawal of neonicotinoids in the EU has been the subject of debate and controversy [86,87]. Our results support that these systemic insecticides show differential toxicity to buff-tailed bumblebees. The neonicotinoid imidacloprid, banned for use in the EU since 2018, caused high mortality in *B. terrestris* workers, while thiacloprid, also currently banned, showed significantly less acute toxicity to this species. Pyrethroids, which are highly probable candidates to replace the banned neonicotinoids, showed intermediate toxicity between the two neonicotinoids, and there was a tendency for bumblebees to decrease their consumption with increasing concentrations. Sulfoxaflor, also a systemic insecticide that has been on the market for only a few years, was found to be the second most toxic insecticide tested, behind only imidacloprid. Taking into account the presence of residues in nectar/honey of these five insecticides, under worst case scenario conditions imidacloprid would have by far the highest risk for this species, followed by sulfoxaflor. Finally, the microbial insecticide formulated from insecticidal toxins of *B. thuringiensis* showed no significant toxicity to this species. 

Therefore, the replacement of neonicotinoids by other types of insecticides for pest control may also have an impact on bee populations as some of the alternatives have already been shown to be toxic not only to *B. terrestris*, but also to other bees. It is vital to strive for a more comprehensive approach to ensure bee health in the broadest sense. To this end, laboratory studies on acute and chronic toxicity need to be complemented by studies under realistic natural conditions to allow a proper risk assessment of insecticides in adults and immatures in managed and wild species [31,32,33].

## Figures and Tables

**Table 1:** Toxicity of insecticides with different mode of action on *B. terrestris* workers after 48 h oral exposure. Data expressed as lethal concentration (LC) or lethal dose (LD).

			Lethal Concentration					Lethal Dose				
Active Ingredient ^a^	N ^b^	d.f.	Slope (SE)	χ^2^	LC_50_ (CI 95%) ^c^	LC_90_ (CI 95%) ^c^	LCR (LC_50_) (CI 95%) ^d^	Slope (SE)	χ^2^	LD_50_ (CI 95%) ^e^	LD_90_ (CI 95%) ^e^	LDR (LC_50_) (CI 95%) ^d^
Imidacloprid (N)	149	23	1.15 (0.23)	28.9	0.38 (0.22–0.76)	4.96 (1.85–58.6)	1103 (608–2003) *	1.2 (0.2)	28.4	0.13 (0.08–0.24)	1.31 (0.55–10.78)	687.3 (352.5–1340.4) *
Thiacloprid (N)	95	8	2.52 (0.64)	9.2	424 (296–815)	1366 (744–15338)	1	1.8 (0.4)	7.1	90.5 (58.8–172.7)	554 (252–4330)	1
Deltamethrin (*p*)	99	8	1.33 (0.32)	5.1	7.1 (3.3–11.9)	64.5 (31.2–382.9)	60.1 (29.9–121) *	1.8 (0.4)	5.4	3.65 (2.19–5.11)	15.5 (9.6–52.8)	24.85 (12.86–47.65) *
Esfenvalerate (P)	191	32	2.68 (0.38)	36.5	17.8 (14.4–22.4)	53.5 (38.3–96.1)	23.8 (16.4–34.6) *	3.2 (0.5)	43.4	5.52 (4.55–6.70)	12.5 (9.4–23.2)	44.21 (12.7–155) *
Sulfoxaflor (S)	126	19	3.73 (0.97)	26.4	2.22 (1.66–3.85)	4.90 (3.15–30.1)	191 (162–289) *	5.6 (1.5)	22.8	0.71 (0.56–1.01)	1.28 (0.93–5.30)	123.3 (72.6–209) *

^a^ (N): neonicotinoid; (P): pyrethroid; (S): sulfoximine. ^b^ Number of *B. terrestris* workers used in the bioassay including the positive and negative controls. ^c^ LC_50_ and LC_90_ and their 95% confidence intervals (CI) expressed in ppm (*W/V*). ^d^ LCR: lethal concentration ratio; LDR: lethal dose ratio. LC_50_ and LD_50_ are significantly different (* *p* < 0.05) if the 95% CI of the of the LCR or LDR do not include the value 1 [56]. ^e^ LD_50_ and LD_90_ and their 95% CI expressed in µg of active ingredient/bee.

**Table 2:** Maximum exposure dose of *B. terrestris* workers to insecticides based on the maximum insecticide residues found in nectar or honey.

	Max. Residue (ppb) ^a^	LD_50_ (µg a.i./Bee)	Max. Exposure Dose (ng a.i./Bee/Day) ^c^	No. Days a Worker Needs to Reach the LD_50_ (Worst Case Scenario) ^d^
Imidacloprid	72.8	0.13	40.3	3.2
Thiacloprid	208.8	90.5	115.7	782.3
Deltamethrin	6.7	3.65	3.7	983.8
Esfenvalerate	0.7 ^b^	5.52	0.39	14,234.1
Sulfoxaflor	13.8	0.71	7.6	92.9

^a^ Maximum insecticide residues detected in nectar or honey for imidacloprid, thiacloprid, deltamethrin, fenvalerate [54] and sulfoxaflor [55]. ^b^ This value corresponds to maximum fenvalerate residue found in honey, as no published data on esfenvalerate residue were found. ^c^ Maximum dose of insecticide consumed per bee and day, assuming that each bee consumes 554 mg of nectar per day (maximum consumption value). ^d^ Number of days a worker needs to reach the LD_50_ after maximum consumption of the highest concentration of insecticide found in residue.

## Data Availability

Not applicable.

## Supplementary Materials

The following supporting information can be downloaded at: https://www.mdpi.com/article/10.3390/insects13020184/s1, Table S1: Number of workers included in the bioassays with insecticides, from each of the commercial colonies used. Only the dose-response bioassays used for subsequent probit analysis are included.
