# Supplementary material for: Sensitivity of Buff-Tailed Bumblebee (Bombus terrestris L.) to Insecticides with Different Mode of Action
Source: Insects. 2022 Feb 10;13(2):184. doi: 10.3390/insects13020184 (PMC8879041; doi:10.3390/insects13020184)
Supplement: Supplementary file 1 [file insects-13-00184-s001.zip › insects-1526892-supplementary.pdf]

**Table S1.** Number of workers included in the bioassays with insecticides, from each of the commercial colonies used. Only the dose-response bioassays used for subsequent probit analysis are included.

| Bioassay | Nº of replicate (colony) | Nº of workers |
|---|---|---|
| Imidacloprid | 1 | 42 |
| | 2 | 26 |
| | 3 | 30 |
| | 4 | 28 |
| | 5 | 23 |
| Thiacloprid | 1 | 38 |
| | 2 | 57 |
| Deltamethrin | 1 | 47 |
| | 2 | 52 |
| Esfenvalerate | 1 | 24 |
| | 2 | 20 |
| | 3 | 32 |
| | 4 | 34 |
| | 5 | 17 |
| | 6 | 28 |
| | 7 | 36 |
| Sulfoxaflor | 1 | 50 |
| | 2 | 26 |
| | 3 | 21 |
| | 4 | 16 |
| | 5 | 13 |
